# Misuse of Guidelines Could Disadvantage and Harm Patients

**DOI:** 10.1111/jebm.12666

**Published:** 2024-12-16

**Authors:** Ernest W Lau, Hendrik Bonnemeier, Benito Baldauf

**Affiliations:** ^1^ Department of Cardiology Royal Victoria Hospital Belfast UK; ^2^ Department of Cardiology University Rostock Rostock Germany; ^3^ Medical Faculty Christian‐Albrechts‐University Kiel Germany; ^4^ Department of Life Sciences University of Applied Science Bremerhaven Germany

1

Guidelines are increasingly regarded as the “reference standards” in clinical decision making. Randomized controlled trials (RCTs) top the evidence hierarchy used in guidelines synthesis. If the RCTs show positive results, their inclusion and exclusion criteria become the treatment's indications (Class I–II). Contrariwise, the characteristics for the subgroups who experience more harm than benefit become the contra‐indications (Class III). RCTs involving human participants could not be designed to show a treatment is ineffective or harmful by the Declaration of Helsinki [1]. All the contra‐indications known for a treatment are from either totally unexpected outcomes from RCTs or rare adverse events that only emerge under long term surveillance.

RCTs were developed to provide reliable objective assessment of the effectiveness of medical treatments [2]. Because most medical treatments are only modestly effective, very large sample sizes and prolonged follow‐up are needed for adequate statistical powers to detect treatment effects, making RCTs prohibitively expensive to run. To maximize the chance of “positive” results, RCTs set very stringent inclusion and exclusion criteria so that only patients most likely to benefit and least likely to suffer harm from the investigated treatments are enrolled. RCTs are mostly funded by commercial companies even if they are conducted by academic institutes and healthcare facilities. The stated reason of many RCTs is to compare the safety and efficacy of treatment options for a disease to help patients. The unstated reason that compels and motivates companies to fund costly RCTs is the legal requirement for regulatory approval before they could market medical products. Benefiting patients and protecting them from harm come as the incidental consequences of companies’ fiduciary duties to their shareholders. The tool (RCTs) used by companies to serve their own interests is “co‐opted” by learned societies for synthesizing guidelines intended to help doctors make clinical decisions. The “misuse” of any tool for unintended purposes would likely result in sub‐optimal outcomes.

While RCTs might excel in “internal validity,” they disappoint in “external validity”—their results might not be generalizable to patient populations outside [3, 4]. About 80% of RCTs excluded ≥50% the patients screened for enrollment [5]. For the excluded (unstudied) patients, the RCTs are “agnostic” on their benefit: risk balance for the treatment (Figure 1a). No evidence for benefit (Figure 1a) is not equivalent to evidence of no benefit (Figure 1b). Many patient groups are under‐represented in RCTs for factors (e.g. age, co‐morbidities, etc.) or protected characteristics (gender, ethnicity, etc.) which may not influence their response to a treatment. A default assumption of no benefit for all patient groups omitted from RCTs would unfairly deprive numerous potential beneficiaries of life‐saving or improving treatments. It would be a gross misuse of guidelines with a huge potential of causing health inequality to justify denial of treatments to patients purely on the basis of no evidence of benefit because they have been “excluded,” “neglected” in RCTs for nonmedical reasons [6]. Moreover, the treatment effects demonstrated in RCTs might not be replicated in patients outside the tightly controlled settings anyway. “Real world evidence” based on “real world data” is needed to confirm the safety and efficacy of treatments “proven” in RCTs in widespread clinical use [7].

**FIGURE 1:**
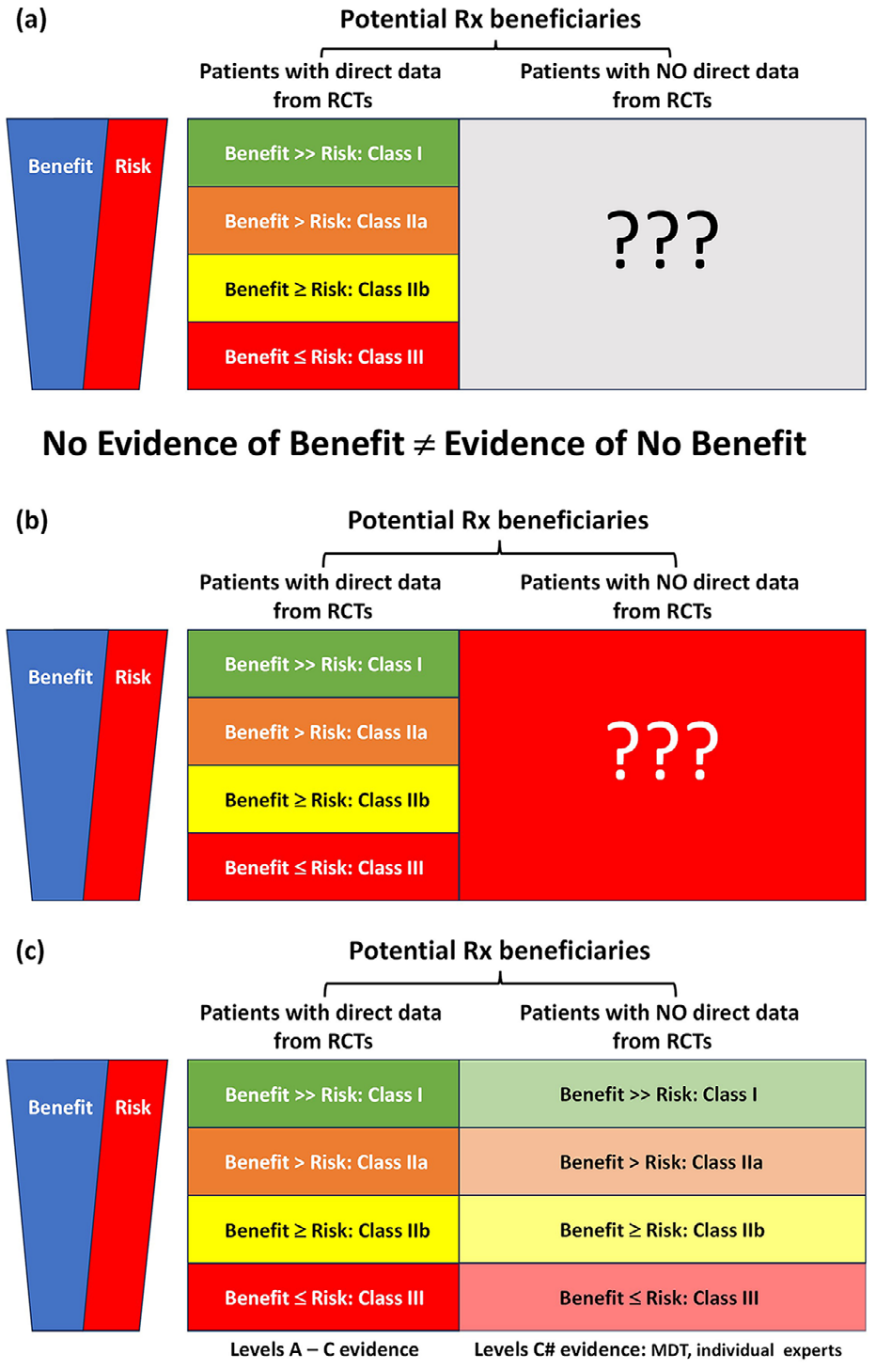
Classes of indications and levels of evidence used in clinical guidelines RCT: randomized controlled trial; MDT: multidisciplinary team.

An indication class in guidelines gives a rough estimate of the “average probability” that a patient would derive more benefit than harm from a treatment, but a good clinical outcome is not guaranteed. Within the same guideline indication class, some patients would benefit more than others, and some patients might even suffer harm rather than benefit. The indication classes listed in guidelines give the false impression that benefit: risk balance of a treatment falls into discrete categories separated by “quantum” leaps. In reality, the benefit: risk balance of a treatment more likely forms a continuum. Guidelines are misused if a patient's likely clinical response and treatment decision are “pigeonholed” into one of these rigid categories like the proverbial “Procrustean bed” [8]. Patients in distinct guidelines indication categories may differ marginally rather than substantially in their benefit: risk balances. Personalized medicine aims to tailor treatments more “precisely” for patients as individuals rather than “generic” members of a cohort [9]. Personalization of treatments could also be assessed in RCTs [10].

The nonequivalence of no evidence of benefit and evidence of no benefit extends to medical conditions so rare that enough patients for RCTs or registries could not be found. In such a situation, “expert opinions” (EO) [11] and “limited data” (LD) from physiological and mechanistic studies might be the only evidence available [12]. The American College of Cardiology and American Heart Association system of recommendation classes EO and LD as the lowest (least reliable) Level of Evidence (LOE) C, with the proviso that:

“A recommendation with LOE C does not imply the recommendation is weak. Many important clinical questions addressed in guidelines do not lend themselves to clinical trials. Although RCTs are unavailable, there may be a very clear clinical consensus that a particular test or therapy is useful or effective.”

The qualification “*a very clear clinical consensus*” is not precisely defined (e.g. how many or what proportion of experts need to agree; how are these experts chosen [11]) and conduces to disagreement. The 2013 version of the Declaration of Helsinki permits a physician to use a nonproven intervention to help a patient when no alternative therapies exist or existent therapies are ineffective, with due informed consent and expert opinions [1]. Without instances of “off‐label” use of nonproven treatments, “*a very clear clinical consensus*” would never emerge. To deny patients access to and denounce doctors for trialing a potentially beneficial but nonproven treatment would be a misuse of guidelines.

LOE C‐EO is based on “expert opinions” [11], which are by definition subjective and hence may vary among different groups/people at any one time and shift within the same group/person over time. The “primacy” of the opinions of the few selected (or self‐selected) experts involved in guidelines synthesis over those held by the much larger pool of their peers not so involved should not be automatic. Doctors not involved in guidelines synthesis should still be allowed to pass their own personal “expert opinions” in assessing the benefit: risk balance of a treatment for patients under their care, forming a new level of evidence LOE C#‐EO (Figure 1c). The merit of an opinion rests on the verity of its premises, validity of its logical deductions, and not the identity or status of its proponents. Doctors managing patients clinically know many nuanced aspects of their medical histories not easily captured in RCTs and reflected in guidelines and may also have fewer conflicts of interest. They also bear full professional and legal responsibilities for their clinical decisions. Patients should be informed of the full spectrum of professional opinions on their management options even if and especially when there is disagreement among doctors.

The indications for a treatment in clinical guidelines typically focus on the characteristics of the patient. However, provider factors (center's volume; operator's experience) could also influence the outcomes to a treatment, especially when the treatment is an invasive intervention. RCTs give the outcomes for an invasive procedure averaged over many centers and operators. The indication classification in clinical guidelines may thus not be reflective of the likely clinical outcome if a patient receives the treatment from a particular provider. High volume centers and operators tend to produce better outcomes than low volume centers and operators, even on frail and challenging patients. Providers’ experience and confidence in their ability to execute an invasive procedure successfully is likely to influence and even determine their willingness to offer the treatment to patients [13]. Providers may exist in competition rather than collaboration and hence are reluctant to cross‐refer even if that would be in the patients’ best interests. Doctors have the duty to inform patients of these caveats about invasive treatments when discussing management options. Clinical guidelines typically do not include provider factors in the indications for a treatment.

Clinical guidelines are not legally binding. While they would be taken into consideration, compliance with and deviation from guidelines would not automatically result in exoneration or punishment by the court [14, 15]. The legal system operates its own standards for determining medical negligence and malpractice. Doctors could and need to exercise judgement in their use of guidelines.

All tools have their proper uses but could produce unintended results if misused. Guidelines are clinical decision support tools to help clinicians improve the outcomes for patients through “better” decisions. However, if a good outcome is the ultimate goal, the decision leading to it cannot be judged on its own without taking into account its execution. A “sound” decision could be ruined by “botched” implementation; a “unsound” decision could be salvaged by “masterful” execution. Rule compliance is a mean to the end (a good clinical outcome) and should not become the end itself. What the patient cares about is the outcome and not the process. Narrow interpretation and rigid application of clinical guidelines without taking into account the many caveats expounded above is a misuse that could disadvantage and even harm patients.

## Conflicts of Interest

The authors declare no conflicts of interest.
